# Ethnomedicines and anti-parasitic activities of Pakistani medicinal plants against *Plasmodia* and *Leishmania* parasites

**DOI:** 10.1186/s12941-016-0170-0

**Published:** 2016-09-20

**Authors:** Akash Tariq, Muhammad Adnan, Rahila Amber, Kaiwen Pan, Sakina Mussarat, Zabta Khan Shinwari

**Affiliations:** 1 Key Laboratory of Mountain Ecological Restoration and Bioresource Utilization & Ecological Restoration Biodiversity Conservation Key Laboratory of Sichuan Province, Chengdu Institute of Biology, Chinese Academy of Sciences, Chengdu, 610041 China; 2 Department of Botany, Kohat University of Science and Technology, Kohat, 26000 Pakistan; 3 Department of Zoology, Kohat University of Science and Technology, Kohat, 26000 Pakistan; 4 Department of Biotechnology, Quaid-i-Azam University Islamabad, Islamabad, 44000 Pakistan

**Keywords:** Ethnomedicines, Malaria, Leishmaniasis, Phytochemicals, In vitro activities

## Abstract

**Background:**

Leishmaniasis and malaria are the two most common parasitic diseases and responsible for large number of deaths per year particularly in developing countries like Pakistan. Majority of Pakistan population rely on medicinal plants due to their low socio-economic status. The present review was designed to gather utmost fragmented published data on traditionally used medicinal plants against leishmaniasis and malaria in Pakistan and their scientific validation.

**Methods:**

Pub Med, Google Scholar, Web of Science, ISI Web of knowledge and Flora of Pakistan were searched for the collection of data on ethnomedicinal plants. Total 89 articles were reviewed for present study which was mostly published in English. We selected only those articles in which complete information was given regarding traditional uses of medicinal plants in Pakistan.

**Results:**

Total of 56 plants (malaria 33, leishmaniasis 23) was found to be used traditionally against reported parasites. Leaves were the most focused plant part both in traditional use and in in vitro screening against both parasites. Most extensively used plant families against Leishmaniasis and Malaria were *Lamiaceae* and *Asteraceae* respectively. Out of 56 documented plants only 15 plants (*Plasmodia* 4, *Leishmania* 11) were assessed in vitro against these parasites. Mostly crude and ethanolic plant extracts were checked against *Leishmania* and *Plasmodia* respectively and showed good inhibition zone. Four pure compounds like artemisinin, physalins and sitosterol extracted from different plants proved their efficacy against these parasites.

**Conclusions:**

Present review provides the efficacy and reliability of ethnomedicinal practices and also invites the attention of chemists, pharmacologist and pharmacist to scientifically validate unexplored plants that could lead toward the development of novel anti-malarial and anti-leishmanial drugs.

## Background

Leishmaniasis and malaria are two most common parasitic diseases and infects a large number of human populations worldwide. Leishmaniasis is endemic disease of almost 88 countries in which about 350 million peoples are at risk of infection [1]. Malaria is a major public health problem throughout the world and causes one million deaths per year particularly in developing countries [2].

Leishmaniasis is caused by an obligate intracellular protozoan parasite of genus *Leishmania* while transmitted to humans and other animals by many species of phlebotomus sand flies [3, 4]. The main causative agents for leishmaniasis are *Leishmania tropica, Leishmania major, Leishmania aethiopica*, *Leishmania donovani* and *Leishmania infantum.* The four main clinical types of leishmaniasis are cutaneous leishmaniasis, mucocutaneous leishmaniasis, diffuse cutaneous leishmaniasis and visceral leishmaniasis. Among these visceral leishmaniasis is very fatal if left untreated. About 90 % cases of visceral leishmaniasis are reported from many countries like Brazil, Bangladesh, Sudan, India and Nepal [5, 6]. Leishmaniasis is a complex group of diseases produces various symptoms in host depending upon parasite’s type [7]. Commonly used allopathic drugs against leishmaniasis are pentavalent antimonials like sodium stibogluconate and meglumine antimoniate etc. These allopathic drugs are mostly unaffordable to the local people and are also not safe due to their toxicity on living system. Many drugs need long term administration to recover from the disease and show side effects depending on the patient’s reaction to medicine [8].

Malaria is caused by an intra-erhytrocytic protozoan parasite of the genus *Plasmodium* and transmitted by female anopheles mosquito [9]. The four main species of *Plasmodia* which infects humans are *Plasmodium falciparum, Plasmodium vivax, Plasmodium knowlesi, Plasmodium malariae,* and *Plasmodium ovale*. Globally the most important species is *P. falciparum* causing severe and potentially fatal malaria [2, 10]. For the treatment of malaria many drugs like chloroquine, halofantrine, pyrimethamine, mefloquine, quinine and artemisinin are used [2, 4]. Many problems like resistance of the parasites to drugs, lack of effective vaccines, resistance of mosquito vectors to insecticides and socioeconomic problems rendering treatment of malaria through chemotherapy ineffective [11, 12].

Leishmaniasis and malaria has become a particular problem in the rural areas of Pakistan of all the provinces [13]. Approximately 66 % people of Pakistan live in rural areas [14] and majority of the rural population in Pakistan is poor and cannot afford such expensive drugs for the treatment of leishmaniasis and malaria [15, 16]. Mostly in rural areas peoples depend on medicinal plants for the treatment of various diseases particularly leishmaniasis and malaria [17]. Traditional medicines are extensively using in Pakistan due to easily affordability and efficacy against various diseases [18, 19].

The present review was designed to gather utmost fragmented published literature on anti-malarial and anti-leishmanial plants used by local people in Pakistan. This review will also provide information on in vitro screening and phytochemical investigation of documented plants against these parasites. Future outcomes of this review are to provide evidences regarding the efficacy and reliability of ethnomedicines against *Leishmania* and *Plasmodia* parasites, identify scientific gaps present in current knowledge and to recommend future research areas for the development of effective anti-malarial and anti-leishmanial drugs with fewer side effects.

## Methods

**Fig. 1:**
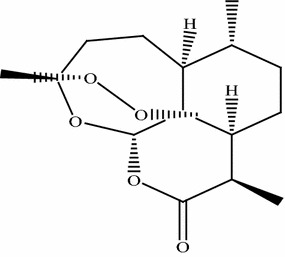
Artemisinin [47]

**Fig. 2:**
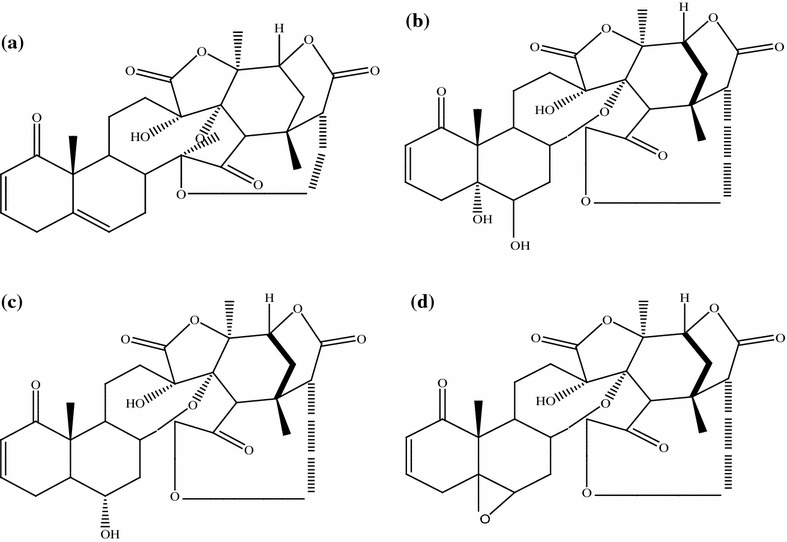
**a** Physalin B [88], **b** physalin D [88], **c** physalin G [88], **d** physalin F [88]

**Fig. 3:**
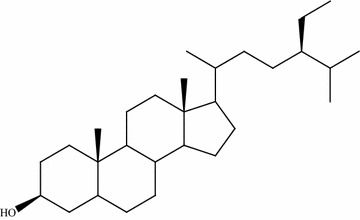
Sitosterol [89]

This review paper was designed by collecting and consulting large number of mostly published literature on medicinal plants used to treat leishmaniasis and malaria in Pakistan. Pub Med, Google Scholar, Web of Science, ISI Web of Knowledge and Flora of Pakistan were searched for the collection of data on ethnomedicinal plants. Plant list and Tropicos were searched for the corrections of plant scientific names, publication authors, synonyms and families. Different search indicators like ethnomedicinal plants used against leishmaniasis and malaria, in vitro activity of different medicinal flora of Pakistan, epidemiology of leishmaniasis and malaria in world, prevalence of leishmaniasis and malaria in Pakistan, drug resistance potential of *Leishmania* and *Plasmodia* parasites were used for the collection of data from the database. Total 89 articles were reviewed for present study which was mostly published in English. We selected only those articles in which complete information was given regarding traditional use of medicinal plants in Pakistan. In-vitro activity of those plants has been mentioned which were checked against *Leishmania and plasmodia* parasites. On the bases of selected data from literature, three tables were formulated using Microsoft Excel 2007 and Microsoft Word 2007. Tables 1 and 2 were formulated on medicinal plants used to treat leishmaniasis and malaria in Pakistan. These tables contains plant name, family, local name, part used, study area and phytochemistry. Table 3 was formulated on anti-parasitic activity of medicinal plants against *L. tropica, L. major* and *P. falciparum.* Concentration of plant’s extract (µg/ml) and their inhibition (%) against parasites were also mentioned. Pure compounds isolated from ethnomedicinal plants and assessed against these parasites have also been mentioned in this review article. Chemical structures of compounds were drawn using ChemDraw software and shown in (Figs. 1, 2 and 3) (CambridgeSoft\ ChemOffice2004\ChemDraw).

**Table 1:** Medicinal plants used to treat leishmaniasis in Pakistan

S. no	Botanical name/family name	Common and or local/name (s)	Part used	Area	Phytochemistry	Citation
1	*Aloe vera* (L.) Burm.f./Xanthorrhoeaceae(= *Aloe barbadense* Mill./Liliaceae)	Kuwargandal, Aloe vera	Leaves	Dera Ismail Khan	Sitosterol	[61]
				Kohat		[25, 57]
2	*Asparagus gracilis* L./Asparagaceae	Shagandal	Aerial parts	Islamabad	Glycosides, tannins, saponins	[22]
3	*Asparagus* *asiaticus* L./Berberidaceae (= *Berberis baluchistanica* Ahrendt.)	Zarch	Roots	Kalat	Alkaloids, flavonoids, saponins, diterpenes, phenols	[62]
4	*Trachyspermum* *ammi* (L.) Sprague/Apiaceae (= *Carumcopticum* L./Umbelliferae)	Ajwain	Whole plant	Quetta	NA	[48]
5	*Citrullus colocynthis* L. Schrad/Cucurbitaceae	Bitter apple, Kortuma	Fruits	Nushki	Ursolic acid, cucurbitacin E 2-0-β-D-glucopyranoside and cucurbitacin I 2-0-β-D-glucopyranoside, alkaloids, flavonoids, saponins, tannins, terpenoids, diterpenes, coumarins	[35]
6	*Juniperus* M.Bieb./Cupressaceae	Juniper	Fresh berries	Ziarat	Alkaloids, flavonoids, saponins, diterpenes, phenols	[37]
7	*Jurinea dolomiaea* Boiss./Compositae	Nazar zela	Roots	Kohistan	Alkaloids, flavonoids, saponins, terpenoids, phenols	[22]
8	*Melia azedarach* L./Meliaceae	Neem, Chinaberry tree, Persian lilac	Green fruits	Islamabad	Phenols	[21]
9	*Nepeta praetervisa* Rech.f./Lamiaceae	Simsok	Leaves	Kalat	Carbohydrate, tannins, phenols, alkaloids, flavonoids, diterpenes, quinones, cardiac glycosides, terpenoids, triterpenoids, coumarins	[63]
10	*Onosma griffithii* Vatke./Boraginaceae	Golden drop	Whole Plant	Malakand	NA	[64]
11	*Perotis hordeiformis*Nees ex Hook. &Arn./Poaceae	Kikuyu grass	Leaves	Soorab	Alkaloids, flavonoids, saponins, diterpenes, phenols	[65]
12	*Physalis minima* L./Solanaceae	Pygmy Ground cherry, Gooseberry	Whole plant	Karachi	Physalins	[66]
13	*Rhazya stricta* Decne./Apocynaceae	Aizwarg	Leaves	Nushki	Alkaloids, flavonoids, saponins, diterpenes, phenols	[34]
14	*Salvia bucharica* Popov./Lamiaceae	Sage, Gul-e-Kakar	Leaves	Quetta	NA	[67]
15	*Sarcococca* *wallichii* Stapf/Buxaceae (= *Sarcococca coriacea* Mull. Arg.)	NA	Roots	Karachi	Steroidal alkaloids	[68]
16	*Sarcococca hookeriana* Baill./Buxaceae	Sweet box	Whole plant	Karachi	Steroidal alkaloids	[66]
17	*Sida cordata* L. (Burm.f.) Borss. Waalk./Malvaceae	Simak	Whole plant	Islamabad	Phenols, saponins, flavonoids	[22]
18	*Stellaria* *media* L. Vill./Caryophyllaceae	Gander	Whole plant	Islamabad	Glycosides, flavonoids, phenols, saponins, terpenoids	[22]
19	*Swertia chirata* Roxb ex./Gentianaceae	Chirata	Seeds	D. I. Khan	Amarogentin, amaroswerin, sweroside	[69]
20	*Tamarix aphylla* (L.) H.Karst./Tamaricaceae	Ghaz, Tamarisk, Salt cedar	Barks	Kohat	NA	[25]
21	*Thuspeinanta brahuica* (Boiss.) Briq./Lamiaceae	NA	Leaves	Kalat	Alkaloids, flavonoids, saponins, phenols, diterpenes	[70]
22	*Tylophora hirsute* Wight/Apocynaceae (= Asclepiadaceae)	Damvel	Aerial parts	Malakand	NA	[71]

*NA* indicates data not available

**Table 2:** Medicinal plants used to treat malaria in Pakistan

S. no	Botanical name/family name	Common and or local name (s)	Area	Part used	Phytochemistry	Citation
1	*Acacia nilotica* L. (Delile)/Leguminosae (= Fabaceae)	Kikar	Mardan	Leaves	Terpenoids	[72]
2	*Ajuga* *integrifolia* Buch.-Ham./Lamiaceae (= *Ajugabracteosa* Wall ex Benth./Labiatae)	Rati buti	Maradori valley	Leaves	NA	[73]
3	*Allium cepa* L./Amaryllidaceae (= Liliaceae)	Piaz	Bannu	Bulb	NA	[74]
4	*Artemisia annua* L./Compositae (= Asteraceae)	Afsantin jari, Sweet Wormwood	Northern areas	Whole plant	Artemisinin	[75]
			Maradori valley	Root		[75]
5	*Artemisia japonica* Thunb./Compositae (= Asteraceae)	Barmar, Basna Tashang	Northern areas	Whole plant	Artemisinin	[75]
6	*Artemisia maritime* L./Compositae (= Asteraceae)	Tarkh, Zoon, Rooner	Northern areas	Whole plant	Artemisinin	[75]
7	*Artemisia scoparia* Waldst. and Kitam./Compositae (= Asteraceae)	Lungi booti	Bhimber	Flowering shoots	Artemisinin	[76]
8	*Azadirachta indica* A.Juss./Meliaceae	Neem	D. I. Khan	Seeds, Leaves	Limonoid (gedunin)	[73]
9	*Bupleurum longicaule* Wall.ex DC./Apiaceae	Proshi	Maradori valley	Root	NA	[73]
10	*Calotropis procera* (Aiton) Dryand./Apocynaceae (= Asclepiadaceae)	Sodom apple, Mudar, Milk weed	Cholistan desert	Root	Alkaloids, Flavonoids, Nitrogen, Crude protein, Crude fiber, Soluble phosphates	[77]
			Karachi			[16]
11	*Capparis spinosa* L./Capparidaceae	Kaveer	Chitral	Flowers	NA	[78]
12	*Trachyspermum* *ammi* (L.) Sprague/Apiaceae (= *Carumcopticum*L./Umbelliferae)	Ajwain	Quetta	Whole plant	NA	[48]
13	*Datura stramonium*L./Solanaceae	Jimson weed	Faisalabad	Leaves	Alkaloids, flavonoids, saponins, glycosides, tannic acid, vitamin C, steroids	[79]
14	*Dodonaea viscosa* (L.) Jacq./Sapindaceae	Ghwarasky	Allai valley	Seeds	NA	[80]
15	*Enicostemma hyssopifolium* Willd./ Gentianaceae	Chhota Chirayata, Nagajihva	Karachi	Whole plant	NA	[16]
16	*Eucalyptus camaldulensis* Dehnh./Myrtaceae	River red gum	Karachi	Leaves, stem	NA	[16]
17	*Fagonia cretica*L./Zygophyllaceae	Azghakey	Mardan	Leaves	Terpenoids	[72]
18	*Helianthus annuus* L./Compositae (= Asteraceae)	Maera stargay gul, Sunflower	Bannu	Leaves	NA	[74]
19	*Melia azedarach* L./Meliaceae	Neem, Chinaberry Tree	Islamabad	Green fruits	Phenols	[19]
20	*Moringa oleifera* Lam./Moringaceae	Sajna, Marango, Moonja	Faisalabad	Whole plant	NA	[81]
21	*Nerium oleander* L./Apocynaceae	Adelfa, Rose bay	Faisalabad	Leaves	Alkaloids	[79]
22	*Origanum majorana* L./Lamiaceae	Sweet marjoram	Faisalabad	Aerial parts	NA	[36]
23	*Origanum vulgare* L./Lamiaceae	Satar, Pot marjoram	Faisalabad	Aerial parts	NA	[36]
24	*Peganum harmala* L./Nitrariaceae (= Zygophyllaceae)	Harmal	Northern areas	Seeds	β-carboline alkaloid (isoharmine), harmaline, harmine	[82]
25	*Polygonatum verticillatum* (L.) All./Asparagaceae	Worlds Solomon’s seal	Swat	Aerial parts	α-Bulnesene, Linalyl, acetate, eicosadienoic, docosane, pentacosane, piperitone	[83]
26	*Psidium guajava* L./Myrtaceae	Amrood	Mardan	Leaves	Terpenoids	[72]
27	*Swertia chirata* Roxb ex./Gentianaceae	Chirata	D. I. Khan	Seeds	Glycosides: Amarogentin, amaroswerin, sweroside	[69]
28	*Swertia paniculata*Wall./Gentianaceae	Momera	Allaivalley	Whole plant	NA	[80]
29	*Tagete sminuta* L./Compositae(= Asteraceae)	Marigold	Northern areas, Abbotabad	Seeds	Terpenoids, saponins, tannins, flavonoids, alkaloids	[45, 84]
30	*Viburnum nervosum*D. Don/Caprifoliaceae (= Adoxaceae)	NA	Azad Jammu Kashmir	Whole plant	Butilinol, oleanolic acid, butilinic acid, urosolic acid,α-amyrin, β-sitosterol	[85]
31	*Vincetoxicum stocksii* Ali &Khatoon/Apocynaceae (= Asclepiadaceae)	NA	Quetta	Whole plant	NA	[48]
32	*Viola odorata* L./Violaceae	Banafsha	Maradori valley	Whole plant	NA	[73]
33	*Xanthium strumarium* L./Compositae (= Asteraceae)	Desi Arinad	Allai valley	Leaves	NA	[80]

*NA* indicates data not available

**Table 3:** In-vitro screening of traditionally used anti-leishmanial and anti-malarial plants against *Leishmania* and *Plasmodia* parasites

Plant name	Part used	Parasite type	Extract	Concentration (µg/ml)	Inhibition (%)	Citation
*Aloe vera*	Leaves	*Leishmania tropica*	Crude methanol	25	15	[25]
				50	27	
				75	43	
				100	66	
*Artemisia annua*	Leaves	*Plasmodium falciparum*	Aqueous	0.095	50	[86]
*Azadiracha indica*	Leaves	*Plasmodium falciparum*	Ethanol	2.4	50	[36]
				2.5	50	
*Asparagus* *asiaticus*	Roots	*Leishmania major*	Crude methanol	25	22	[63]
				50	34	
				250	42	
				500	51	
			Amphotericin B (Reference drug as a control)	25	50	
				50	75	
				250	88	
				500	100	
*Citrullus colocynthis*	Fruits	*Leishmania major*	Crude methanol	25	67	[35]
				50	71	
				250	88	
				500	100	
*Juniperus excels*	Fresh berries	*Leishmania major*	Crude methanol	25	49	[37]
				50	58	
				250	88	
				500	97	
*Melia azedarach*	Fruit	*Leishmania tropica*	Aqueous	1500	55.9	[86]
				2500	67.4	
				5000	80.4	
*Moringa oleifera*	Leaves	*Plasmodium falciparum*	Acetone	400	59.8	[31]
*Nepeta praetervisa*	Leaves	*Leishmania major*	Methanol	25	39	[62]
				50	54	
				250	68	
				500	78	
*Peganum harmala*	Seeds	*Plasmodium falciparum*	Ethanol	12.5	91	[58]
				25	97.4	
				50	98.5	
				100	99.8	
			Chloroquine	NA	99.6	
*Perotis hordeiformis*	Leaves	*Leishmania major*	Methanol	25	47	[65]
				50	58	
				250	70	
				500	80	
			Amphotericin B (Reference drug as a control)	25	50	
				50	75	
				250	88	
				500	100	
*Rhazya stricta*	Leaves	*Leishmania major*	Crude methanol	25	65	[34]
				50	70	
				250	92	
				500	100	
*Salvia bucharica*	Leaves	*Leishmania major*	Crude methanol	25	44	[67]
				50	40	
				250	59	
				500	75	
*Tamarix aphylla*	Barks	*Leishmania tropica*	Crude methanol	25	20	[25]
				50	28	
				75	54	
				100	84	
*Thuspeinanta brahuica*	Leaves	*Leishmania major*	Crude methanol	25	40	[87]
				50	58	
				75	70	
				100	82	
			Amphotericin B (Reference drug as a control)	25	50	
				50	75	
				75	88	
				100	100

## Medicinal plants used to treat leishmaniasis in Pakistan

Leishmaniasis is a neglected tropical disease. Visceral leishmaniasis and cutaneous leishmaniasis are the two main clinical types of leishmaniasis widespread in Pakistan. The incidence of visceral leishmaniasis has been reported from Dera Ismail Khan, Quetta, Tank, Hazara Division, Northern areas and Azad Jammu Kashmir [20]. About 90 % cases of cutaneous leishmaniasis have been reported from all the provinces of Pakistan [21]. The reported endemic areas of cutaneous leishmaniasis are Dir, Chitral, Swat, Mansehra, Dera Ghazi khan, Gilgat, Skardu, Abbotabad, Azad Kashmir, Chilas, Rawalpindi, Khuzdar, Jacobabad, Lasbela, Derabughti, Rajanpur, Quetta, Qila Saifullah, Qila Abdullah, Pishan, Dera Ismail Khan, Larkana and Dadu [22]. These areas are foot hills of mountainous range and situated in North, South and South-Western Pakistan covering about all the provinces including Azad Kashmir. Growth and development of vector sandfly is promoted by the environmental conditions of these endemic areas [23].

Most of the above mentioned regions of Pakistan are rural in nature lacking modern health and education facilities and inhabitants of these regions have low economic status due to least income sources. Moreover, rural people rely on their rich traditional knowledge for their primary health care due to high cost of allopathic drugs [19]. Present review showed that traditional people use 23 medicinal plants belonging to 19 families for the treatment of leishmaniasis (Table 1). Other areas of Pakistan are also known for containing variety of medicinal plants and classic traditional healing practices but scientific documentation has not been yet done. The most widely used plant families for the treatment of leishmaniasis in Pakistan are *Lamiaceae* (four plants), *Liliaceae* (two plants) and *Asclepiadaceae* (two plants). The family *Lamiaceae* and *Liliaceae* usually ranks high in ethnomedicinal studies not only in Pakistan but throughout the world [24–26]. Perez et al. [27] also reported high number of plants belonging to *Lamiaceae* family having anti-parasitic activity including leishmaniasis. Present findings indicate that *Lamiaceae* family contains variety of anti-parasitic secondary metabolites and should be given focus in future studies. Other reasons of its wide use might be due to higher abundance of these plants in different regions and strong traditional beliefs [28–30]. Almost all plant parts are found to have anti-leishmanial activity but most preferred parts in Pakistan are leaves, fruits, roots and aerial parts. Leaves are also the most focused part of plant in in vitro screening against leishmaniasis not only in Pakistan but other countries of world [31, 32]. Most of the metabolic processes take place in leaves result in production of different secondary metabolites; therefore, it might be attributed with its wider utilization for in vitro screening and traditional medicines [33]. In some areas like Quetta, Islamabad and Malakand whole plant is used to treat leishmaniasis which is major threat to the conservation status of these medicinal plants. People should be educated regarding proper harvesting of these valuable anti-leishmanial plants for sustainable utilization.

## In-vitro activities of anti-leishmanial plants

Majority of the modern allopathic drugs of the world have developed on the basis of traditional knowledge of the people regarding medicinal plants. Among 23 medicinal plants used against leishmaniasis in Pakistan only 11 plants have been studied worldwide for their in vitro activity against *L. major* and *L. tropica* parasites and documented in the present review. Anti-leishmanial activity of medicinal plants has shown excellent activity against *Leishmania* parasite (Table 3). Different plant parts have been used for extract formation experimentally among which leaves, fruits and roots are most widely used parts. This result gives an indication about the reliability of traditional ethnomedicinal knowledge and efficacy of these practices. Different plant extracts like crude methanol and methanol have been used at different concentrations (µg/ml) for their efficacy against *L. major* and *L. tropica* but crude methanol extract is most commonly used [34]. Crude methanol extraction of plant parts is also practiced in other parts of the world. Crude methanolic extract of different plants have shown strong inhibition zone ranges from 65 to 100 % at different concentrations ranging from 25 to 500 µg/ml against *L. major* parasite [34, 35], while methanolic extract of different plant parts having concentration of about 25–500 µg/ml shown optimum inhibition zone ranging from 39 to 80 %. Aqueous extract of a plant have shown inhibition zone ranging from 55.9 to 80.4 % at concentration of about 25–5000 µg/ml [35, 36]. Plant extracts have been proved more effective against leishmaniasis as compared to allopathic drugs due to less toxicity [36]. Therefore it is imperative to investigate and explore medicinal plants scientifically for the development of novel anti-leishmanial drugs of strong efficacy. Experimental investigation of different plants have shown presence of phytochemical constituents such as alkaloids, flavonoids, carbohydrates, diterpenes, saponins, phenols and tannins that might be responsible for their inhibitory activities against *Leishmania* parasites [36, 37]. Very few studies conducted on the purification of pure compounds from above mentioned plants and should be given focus in future.

## Medicinal plants used to treat malaria in Pakistan

In modern medical terms, malaria can be defined as infection caused by red blood cells parasite belonging to genus *Plasmodium*. Malaria is a major serious public health problem caused by *P. falciparum* and *P. vivax*, the two most prevalent *Plasmodium* species throughout the world. Approximately 64 % cases of malaria caused by *P. vivax* and about 36 % cases by *P. falciparum* in Pakistan [38]. According to WHO report about 1.6 million cases of malaria were reported in endemic areas per year [39]. The cases of malaria infection are reported from Sindh, Punjab, Khyber Pakhtunkhwa, Baluchistan and FATA areas. In these regions malaria often occurs in poor people because majority of population in these regions are rural with very low socioeconomic status. The environment of these areas promotes optimum growth of female Anopheles vector [40]. Reason behind high prevalence of malaria in poor people of Pakistan might be due to that malaria strike in the season when economic conditions are more difficult for the people. In Brazil 99 % malarial cases are reported and transmitted in Amazon region, where population consists of tribal people and immigrants from other regions [40]. History has proven traditional medicines to be the best source of effective anti-malarial e.g. *Cinchona* spp. and *Artemisia annua* L. [41].

Chloroquine is the most commonly used antibiotic for the treatment of malaria not only in Pakistan but throughout the world [38]. Low income status of poor people and emergence of antibiotic resistance of parasite encourage the use of traditional medicines for the treatment of malaria. Ethnomedicinal used of plants are common throughout the world including Pakistan [42]. Present review documented 33 anti-malarial plants traditional being use in Pakistan (Table 2). The most widely used plant families for the treatment of malaria in Pakistan are *Asteraceae* (9 plants), *Gentianaceae*, *Lamiaceae* and *Asclepiadaceae* (3 plants each). The medicinal plants belonging to these families are extremely used for medicinal purposes including anti-malarial purposes not only in Pakistan but throughout the world [43–45] that might be due to greater availability or high traditional values of these plants in different regions. Traditional healers mostly used leaves for the preparation of ethnomedicinal recipes against malaria and these findings are not surprising because leaves are the most focused plant part throughout the world [44, 46]. In different regions of Pakistan mostly whole plant is used for the treatment of malaria due to the presence of important compounds. It is considered to be one of the major causes of extinction of highly valuable medicinal plants in many areas of Pakistan.

## In-vitro activities of anti-malarial plants

In present review, among 33 medicinal plants used to treat malaria in Pakistan, only 4 plants have been investigated experimentally throughout the world for their in vitro activity against *P. falciparum* (Table 3). Only two plant parts, seeds and leaves have been used for extract preparation. Different plant extracts like acetone, aqueous and ethanol have been used scientifically at different concentrations (µg/ml) for their efficacy against *P. falciparum* [32, 37, 47]. Ethanol extraction of plants is also followed throughout the world due to its polar nature [42]. Ethanolic extracts of two different plants like *Azadiracha indica* and *Peganum harmala* have shown strong inhibition zone ranging from 50 to 99.8 % at concentration of about 2.4–100 µg/ml against *P. falciparum.* Acetone extract of *Artemisia annua* show 50 % inhibition zone at concentration of about 0.095 µg/ml and aqueous extracts of *Moringa oleifera* show optimum inhibition zone 59.8 % at concentration of 400 µg/ml. These results show the strong efficacy of plants extracts against *P. falciparum* in comparison with standard drug. Phytochemical screening of different plant extracts have not been studied in detail but experimentally studied plant parts mostly contain alkaloids, flavonoids, saponins, tannins and terpenoids that might be responsible for anti-parasitic activities of these plants. Other plants needs in vitro exploration and phytochemical screening that could lead toward extraction of some novel compounds/drugs against *Plasmodium* parasite.

## Medicinal plants with both anti-leishmanial and anti-malarial potential

Three plants *Melia azedarach* L. (*Meliaceae*), *Vincetoxicum stocksii* L. (*Asclepiadaceae*) and *Carum copticum* L. (*Umbelliferae*) have been used for the treatment of both leishmaniasis and malaria which show their high potential for anti-parasitic activity [19, 48]. But in vitro activity of only one plant *M. azedarach* has been investigated experimentally against *Leishmania* parasite. Aqueous extract of fruit of *M. azedarach* showed strong inhibition zone of about 55.9, 67.4 and 80.4 % against *L. tropica* at three different concentrations 1500, 2500 and 5000 µg/ml, respectively (Table 3). Present finding is very interesting because it gives an indication about strong efficacy of these candidate medicinal plants for future research against malaria.

## Active phyto-compounds against *Leishmania* and *Plasmodia* parasites

Only three compounds isolated from traditionally used anti-leishmanial and anti-malarial plants of Pakistan were investigated for their anti-parasitic activity.

### Artemisinin

In present review four plant species of *Artemisia* used in Pakistan to treat malaria (Table 2). The genus *Artemisia* has great importance in pharmaceutics as it is used in traditional medicines to treat various diseases especially malaria not only in Pakistan but throughout the world [49–51]. In-vitro study of *Artemisia* plants shows that they contain an important chemical compound Artemisinin, Sesquiterpenoid lactone. Artemisinin (Fig. 1) is extracted from the leaves of *Artemisia* and known to have best antimalarial activity. WHO recommended the Artemisinin combination therapy for the treatment of malaria caused by *P. falciparum* [2, 52]. *Artemisia annua,* a good source of Artemisinin is endemic plant of China and used as folk medicine to treat malaria for about 2000 years [52]. Artemisinin have also been reported for good anti-viral, anti-cancer and anti-leishmanial activity [53].

### Physalins

Several physalins (Steroidal lactone) were isolated from various species of genus *Physalis* belonging to the family Solanaceae. Physalins (Fig. 2) have both anti-leishmanial and anti-malarial potential [54, 55]. Four types of physalins B, D, G and F (Fig. 2a–d) were isolated from *Physalis angulata*. The in vitro and in vivo activity of physalins B and F showed potent anti-leishmanial activity against various *Leishmania* parasites like *L. brazillenesis, L. amazonensis, L. major* and *L. chagasi* [55, 56]. Physalins B, D, G and F have also been reported for their anti-malarial activity against *P. falciparum* [54].

### Sitosterol

*Aloe barbadense* is an important medicinal plant having bioactive compounds reported for their anti-leishmanial activity. Sitosterol (Fig. 3) is an important compound extracted from the leaves of *Aloe vera*. It inhibits the growth of promastigotes of *L. donovani*, a causative agent for life threatening visceral leishmaniasis disease. The active components of *Aloe vera* target the CDC42 protein in comparison with a natural inhibitor Sacramine B [57].

## Antibiotic resistance of *Leishmania* and *Plasmodia* parasites

Literature review showed that both *Leishmania* and *Plasmodium* parasites have shown resistance to various antibiotics that are being used for the treatment of leishmaniasis and malaria. Various antibiotics have been used for the treatment of malaria worldwide like chloroquine, halofantrine, pyrimethamine, mefloquine, quinine and artemisinin [2, 4]. The in vitro investigation of chloroquine showed resistance range from 69.8 to 99.6 % against *P. falciparum* [58, 59].

The most commonly used drugs for the treatment of leishmaniasis are pentavalent antimonials like sodium stibogluconate and meglumine antimoniate. Beside these many other drugs like amphotericin, ambisome (lipid formulation of amphotericin), miltefosine (impavido), pentamidine and paromomycin were discovered to treat leishmaniasis [60]. All types of *Leishmania* parasites show resistance against one drug or other [19, 60]. Due to emerging potential of drug resistance of parasites, high cost of allopathic drugs and their side effects encourages the use of traditional medicines among local population worldwide.

## Conclusions and future recommendations

Pakistan has tremendous potential regarding the use of ethnomedicines for the treatment of multiple diseases including malaria and leishmaniasis. This review provides a scientific rationale for the traditional uses of medicinal plants against these diseases. Traditional healers of different regions have strong knowledge to utilize medicinal plants. In-vitro screening of traditionally used anti-parasitic plants have proven the efficacy of such plants. Crude plant extracts, methanolic and other extracts were effective in antimalarial and anti-leishmanial activities. Mostly, leaves of documented plants are traditionally used and also for in vitro screening. Different classes of compounds exist in the documented plants including alkaloids, flavonoids and terpenoids. Very few compounds have so far been isolated from the documented plants and tested in vitro against studied leishmanial and malarial parasites. In pure compounds, ursolic acid and cucurbitacin in *C. colocynthis* while glycosides and alkaloids isolated from *R. stricta* possess anti-leishmanial activities. On the other side, compounds such as limonoids (gedunin) from *A. indica* while β-carboline alkaloids, Harmaline and Harmine isolated from *P. harmala* have proven in vitro and in vivo anti-plasmodial activities. Hence, these plant species must be explored for the identification of more such compounds to be used against *Leishmania* and *Plasmodium* parasites. Moreover in the present era, parasites are showing resistance to common allopathic drugs, while on the other side medicinal plants have proven their effectiveness as anti-parasitic drugs. It is therefore imperative to conduct future studies on the unexplored documented plants both in vitro and in vivo for the development of novel drugs. On the basis of findings in this review, following recommendations are suggested:

- Ethnomedicinal studies provide baseline information for future scientific research, therefore it is recommended to expedite exploration of anti-parasitic plants not only in Pakistan but throughout the world.
- Traditional healers mostly use *Lamiaceae* and *Asteraceae* families for the treatment of leishmaniasis and malaria, respectively. It invites the attention of worldwide researchers to explore species belonging to these families both phytochemically and pharmacologically.
- Among all plant parts, leaves have taken more focus both in traditional medicines as well as in in vitro studies. Other plant parts should also be brought under the spotlight for the potential discovery of different compounds.
- Different extracts of documented plants have been used worldwide against leishmaniasis and *Plasmodia*. Crude extracts have been given more preference against leishmaniasis while only very few plants have been tested in vitro against *Plasmodia*. Extracts like methanolic, ethanolic, n-hexane should also be valued against both parasites that could be helpful in extraction of some novel compounds.
- More attention should be given towards the isolation of pure compounds from these plants, and their in vitro investigations against leishmanial and malarial parasites.
- In-vivo studies should also be brought under the focus in order to pharmacologically validate these traditional plants.
- Action mechanism of different extracts and pure compounds on the studied parasites should also be studied in future research.
- Toxicity of these plants should also be tested on living system that would be helpful in proving the reliability of traditional medicines.

## Abbreviations

WHO: World health organization
FATA: Federally Administered Tribal Areas
